# Two-Year Outcome of Selective Laser Trabeculoplasty for Normal-Tension Glaucoma in Japan: First-Line or Second-Line Selective Laser Trabeculoplasty (FSS) Study

**DOI:** 10.3390/jcm14103459

**Published:** 2025-05-15

**Authors:** Tomoko Naito, Koji Nitta, Takako Miki, Akiko Narita, Tairo Kimura, Yasushi Ikuno, Shiro Mizoue, Maki Katai, Yoshiaki Saito, Mami Nanno, Naoki Tojo, Naoto Tokuda, Shigeki Yamabayashi, Katsuyoshi Suzuki, Kimihito Konno, Hiroaki Ozaki, Toru Nakazawa, Tadashi Nakano, Kenji Nakamoto, Naoya Nezu, Shigeru Mori, Kazuyuki Hirooka, Itaru Kimura, Takeshi Sagara, Toyoaki Tsumura, Aika Tsutsui, Kae Sugihara, Takuji Matsuda, Yoshitaka Tasaka, Satoru Tsuda, Tomoyuki Watanabe, Naka Shiratori, Yutaro Tobita, Kaori Komatsu, Akiko Harano, Kazuhisa Sugiyama, Keiji Yoshikawa, Masaki Tanito

**Affiliations:** 1 Grace Eye Clinic, Okayama 700-0821, Japan; 2 Fukui-Ken Saiseikai Hospital, Fukui 918-8503, Japan; nitta.koji7001@fukui.saiseikai.or.jp; 3 Okayama Saiseikai General Hospital, Okayama 700-8511, Japan; 4 Ueno Eye Clinic, Tokyo 110-0015, Japan; 5 Ikuno Eye Center, Osaka 532-0023, Japan; 6 Department of Ophthalmology, Minami-Matsuyama Hospital, Matsuyama 790-0952, Japan; 7 NTT Medical Center Sapporo, Sapporo 060-0061, Japan; makity_k@yahoo.co.jp; 8 Saito Eye Clinic, Kanazawa 920-0867, Japan; 9 Kagurazaka Minamino Eye Clinic, Tokyo 162-0825, Japan; 10 Oyama Eye Clinic, Toyama 930-0194, Japan; 11 Department of Ophthalmology, St. Marianna University School of Medicine, Kawasaki 216-8511, Japan; 12 Yamabayashi Eye Clinic, Nagoya 464-0850, Japan; 13 Suzuki Eye Clinic, Yamaguchi 755-0155, Japan; 14 Hachioji You Eye Center, Tokyo 194-0021, Japan; 15 Department of Ophthalmology, School of Medicine, Fukuoka University, Fukuoka 814-0180, Japan; 16 Department of Ophthalmology, Tohoku University Graduate School of Medicine, Sendai 980-8574, Japan; 17 Department of Ophthalmology, Jikei University School of Medicine, Tokyo 105-8471, Japan; tnakano@jikei.ac.jp (T.N.);; 18 Department of Ophthalmology, Nippon Medical School, Tokyo 113-8603, Japan; user902850@aol.com (K.N.);; 19 Department of Ophthalmology, Tokyo Medical University, Tokyo 160-0023, Japan; 20 Mori Ophthalmic and Medical Clinic, Nagasaki 854-0025, Japan; 21 Department of Ophthalmology and Visual Science, Hiroshima University, Hiroshima 734-8551, Japan; 22 Department of Ophthalmology, Tokai University Hachioji Hospital, Tokyo 192-0032, Japan; 23 Sagara Eye Clinic, Hagi 758-0021, Japan; 24 Fussa Hospital, Tokyo 197-8511, Japan; 25 Department of Ophthalmology, Shimane University Faculty of Medicine, Izumo 693-8501, Japantanito-oph@umin.ac.jp (M.T.); 26 Department of Ophthalmology, Kanazawa University Graduate School of Medical Science, Kanazawa 920-8641, Japan; 27 Yoshikawa Eye Clinic, Tokyo 194-0021, Japan

**Keywords:** selective laser trabeculoplasty, normal-tension glaucoma, first-line treatment, second-line treatment, intraocular pressure, central corneal thickness

## Abstract

**Objectives**: The objective of this study is to investigate the two-year continuous efficacy, risk factors, and safety profile of selective laser trabeculoplasty (SLT) in Japanese individuals diagnosed with normal-tension glaucoma (NTG) who underwent SLT as either a first-line or second-line treatment. **Methods**: A retrospective chart review was conducted of patients with NTG who underwent SLT as either an initial or secondary therapy at 26 medical institutions in Japan between January 2020 and December 2021 with a 2-year follow-up. The primary endpoint was a reduction in the rate of intraocular pressure (IOP) over 2 years after SLT. To estimate the time-varying effect of IOP reduction, a linear mixed-effects model was employed. The secondary endpoints were numerical IOP reduction, treatment success rates shown by a Kaplan–Meier analysis, and complications. Success was defined as an outflow pressure improvement rate (ΔOP) ≥ 20% (definition A) or an IOP reduction rate ≥ 20% (definition B) without further treatment. A Cox proportional hazards regression analysis was used to identify the risk factors to successful SLT treatment. The study was registered with UMIN-CTR (ID: UMIN R000064045). **Results**: A total of 230 eyes from 230 individuals participated in this study, with 148 eyes receiving SLT as an initial (first-line) therapy and 82 eyes undergoing SLT as a secondary (second-line) intervention. In the first-line group, the mean IOP dropped from 16.7 ± 2.3 mmHg to 13.7 ± 2.4 mmHg at two years post-treatment, reflecting a 16.8% reduction. In the second-line group, the average IOP declined from 15.9 ± 2.5 mmHg to 13.2 ± 2.0 mmHg, marking a 14.4% decrease over the same period. The treatment success rate according to definition A (ΔOP ≥ 20%) was 73.7% at 2 years. Analysis using a linear mixed-effects model identified time (*p* < 0.001), age (*p* = 0.044), baseline IOP (*p* < 0.001), and central corneal thickness (CCT) (*p* < 0.001) as statistically significant contributors to IOP reduction following SLT. However, neither the Group (first-line vs. second-line) variable (*p* = 0.386) nor the Time × Group interaction (*p* = 0.298) reached statistical significance. A lower baseline IOP and a thicker CCT were confirmed as significant predictors of SLT treatment failure. **Conclusions**: Both initial and secondary SLT treatments for NTG proved to be effective and safe over a two-year period, although the extent of IOP reduction was smaller in cases with a lower baseline IOP. Our findings indicate that the IOP-lowering effect of SLT in NTG is influenced by pretreatment IOP levels, aligning with previous studies on primary open-angle glaucoma and ocular hypertension. However, in contrast to those earlier findings, our research identified pretreatment central corneal thickness as a statistically significant factor influencing SLT efficacy in NTG. These results support the role of SLT as a reliable and safe therapeutic option for managing NTG.

## 1. Introduction

Lowering intraocular pressure (IOP) is currently the only evidence-based and effective way to prevent glaucoma progression [1,2,3,4,5,6,7,8,9,10,11,12]. One well-established treatment for lowering IOP is selective laser trabeculoplasty (SLT). Findings from the Laser in Glaucoma and Ocular Hypertension (LiGHT) trial [13,14,15,16,17] demonstrated that SLT is a safe and effective first-line therapy for primary open-angle glaucoma (POAG) and ocular hypertension (OHT), and its use was associated with a lower likelihood of requiring surgical intervention. Ang et al. [18] found that the group using IOP-lowering eye drops experienced a higher rate of ocular side effects, including conjunctival hyperemia and eyelid erythema, compared to the SLT group. SLT is an intervention that is not affected by a patient’s adherence [13,14,15,16,17], which can be an improvement over the issue of poor adherence with glaucoma medications. From a cost perspective, SLT was more cost-effective than IOP-lowering eye drops over 3 years in the LiGHT trial [13]. Based on these findings, glaucoma treatment guidelines in Western countries have been updated to recommend SLT as a first-line therapy.

Wong et al. [19] reported that the efficacy of SLT appeared to vary among different types of glaucoma as a result of systematic review and meta-analysis on the efficacy of SLT in open-angle glaucoma (OAG). The mean IOP reduction after SLT was reported to range from 6.9% to 35.9% at one year, with lower efficacy observed in normal-tension glaucoma (NTG) and greater efficacy in pseudoexfoliation syndrome [19]. It has also been reported that a higher baseline IOP was associated with a greater IOP reduction after SLT [20]. Therefore, SLT may be more suitable for treating POAG or exfoliation glaucoma than NTG, as its IOP-lowering effect tends to be greater in eyes with a higher baseline IOP.

NTG is a subtype of POAG where IOP consistently remains within the normal range despite the development and progression of glaucomatous optic neuropathy [21], and in individuals over 40 in Asia, it constitutes the majority of POAG cases. In the Tajimi study from Japan, 92% of POAG patients had NTG [22], while the Namil study from Korea reported 77% [23]. To our knowledge, only two studies have reported on the long-term efficacy and safety of SLT in NTG patients, showing that a significant gap in the literature remains. Nitta et al. [24] reported that IOP significantly decreased from 15.8 mmHg to 13.2 mmHg at 1 year (IOP reduction rate: 15.8%) and 13.5mmHg (12.7%) at 3 years after an SLT performed as a first-line treatment in 42 eyes of 42 NTG patients [24]. Lee et al. [25] showed notable reductions in both IOP and the usage of medication over a two-year period following SLT in 34 patients treated for NTG.

We previously published findings from a prospective cohort study that investigated the 1-year efficacy and safety of first-line or second-line SLT in NTG patients, using IOP reduction rates measured at three-month intervals to evaluate treatment effectiveness [26]. In this study, we extended the follow-up period to two years to evaluate the sustained efficacy of SLT and to analyze the relationship between various covariates and treatment outcomes, an analysis not possible in the previous study. To enhance the reliability of our findings, we also increased the sample size by adding 130 new eyes to the original 100, resulting in a total of 230 eyes.

## 2. Materials and Methods

### 2.1. Study Design

This retrospective, multicenter observational study was conducted at 26 medical institutions across Japan. Patients diagnosed with NTG who underwent either first-line or second-line SLT at any of the participating centers between January 2020 and December 2021 were considered eligible. Detailed descriptions of the inclusion and exclusion criteria, as well as the SLT procedure, are described below. Patients were included if they met the following criteria: a diagnosis of NTG; age of 20 years or older; IOP ≥ 14 mmHg in at least one of the three most recent measurements; a mean deviation (MD) ≥ −15 dB; and a central corneal thickness (CCT) between 450 and 600 μm. The first-line SLT group was comprised of patients who were either newly diagnosed or had discontinued a single-component IOP-lowering eye drop due to allergic reactions. Patients who had discontinued eye drops due to allergic reactions were included in the first-line SLT group only if at least one month had passed since discontinuation and a sufficient washout period was considered to have been achieved. The second-line SLT group included patients who elected to undergo SLT because their current single-component medication (either a prostaglandin analogue or a β-blocker) had failed to achieve sufficient IOP reduction (defined as a decrease < 15% from the baseline).

In the first-line group, all IOP-lowering medications had been discontinued for at least one month prior to SLT. In the second-line group, the concurrent use of eye drops was limited to prostaglandin analogues or β-blockers. Patients were excluded if they had used more than one type of IOP-lowering eye drop (fixed combination drops were counted as two components), received steroid treatment within one month, undergone a sub-Tenon’s triamcinolone acetonide injection within six months prior to SLT, or had a history of laser therapy, refractive surgery, or intraocular surgery (except for cataract surgery performed more than three months before the SLT).

If both eyes of a patient were eligible, only the eye treated first was included in the analysis. All participants provided written informed consent before study enrollment. The SLT was performed using the Tango Ophthalmic Laser system (Ellex Medical, Adelaide, Australia), a Q-switched Nd:YAG laser. Laser parameters included a spot size of 400 μm, a pulse duration of 3 nanoseconds, and a wavelength of 532 nm. Under topical anesthesia, the laser was applied to 360 degrees of the trabecular meshwork using a gonioscope. Laser spots were placed closely without being stacked, using the lowest energy level at which bubble formation just begins to occur.

This study adhered to the principles outlined in the Declaration of Helsinki and received approval from the Medical Research Ethics Committee of the Shimane University Faculty of Medicine (IRB ID: KT20220224-1).

### 2.2. Data Collection and Outcome Measures

A retrospective chart review was performed using medical records from the 26 participating institutions. Baseline data collected prior to SLT included patient age, gender, IOP measured by Goldmann applanation tonometry, visual field results obtained using the Humphrey Field Analyzer with the Swedish Interactive Threshold Algorithm standard and 30-2 or 24-2 programs (Carl Zeiss Meditec, Dublin, CA, USA), refraction, decimal visual acuity, CCT, endothelial cell density measured by specular microscopy, and gonioscopic findings. Follow-up IOP measurements were recorded at 1, 3, 6, 9, and 12 months after SLT, and every 6 months thereafter. Adverse events were documented for all treated eyes.

The primary outcome was the IOP reduction rate, calculated as [(IOP before SLT − IOP after SLT)/(IOP before SLT) × 100] over a 2-year period, analyzed using a linear mixed-effects model. This model was chosen to examine the changes in outcomes over time and their association with risk factors, providing insight into how explanatory variables influence the dependent variable in the longitudinal observational data. Patient ID was included as a random effect, while visit timepoints after SLT, SLT type, age, baseline IOP, CCT, and the interaction term between visit timepoints and SLT type were incorporated as fixed effects. The primary dependent variable was the visit timepoints after SLT, with the interaction term included to account for the effect of SLT type on the relationship between visit timepoints and IOP reduction rate.

The secondary endpoints included the following: numerical reduction in IOP, survival analysis using the Kaplan–Meier method, and the identification of risk factors for SLT treatment success through a Cox proportional hazard regression. In the Kaplan–Meier survival analysis, success was defined by either a ≥ 20% improvement in outflow pressure (ΔOP) (definition A) or a ≥ 20% reduction in IOP (definition B), without the need for additional IOP-lowering medications, a repeat SLT, or other glaucoma surgeries. Cataract surgery was treated as a censoring event due to its potential effect on IOP reduction. ΔOP was calculated using the formula: [(IOP before SLT − IOP after SLT)/(IOP before SLT − episcleral venous pressure (EVP)) × 100], where EVP was assumed to be 10 mmHg. The safety endpoint focused on complications, which included transient IOP spikes (defined as an increase > 5 mmHg from the baseline IOP), anterior chamber inflammation, conjunctival hyperemia, and visual impairment resulting from the SLT.

### 2.3. Statistical Analyses

Statistical analyses were performed using R 4.4.1, with the lme4, lmerTest, and survival packages. No imputation was used for missing data. Continuous variables were compared using a Welch Two Sample *t*-test or a paired *t*-test, depending on the data type, while nominal variables were analyzed with Fisher’s exact test, and ordinal variables were assessed using the Wilcoxon rank sum test. A linear mixed-effects model was applied to examine the IOP reduction rate over the 2-year period following SLT. Cumulative treatment success rates were calculated using Kaplan–Meier survival analysis. Risk factors for treatment success were identified using Cox proportional hazards regression models. A *p*-value of less than 0.05 was considered statistically significant.

## 3. Results

### 3.1. Study Population and Baseline Characteristics

**Figure 1:**
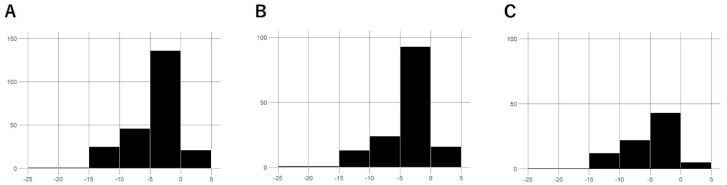
The distribution of mean deviations. (**A**) All, (**B**) first-line group, (**C**) second-line group.

In our previous study [26], 100 eyes from 100 Japanese patients with NTG were included in the analysis. For the current study, we added 130 eyes from 130 new Japanese patients, bringing the total to 230 eyes from 230 patients: 148 eyes/patients in the first-line SLT group and 82 eyes/patients in the second-line SLT group (Table 1). Statistically significant differences were observed between the two groups in terms of mean age, pretreatment IOP, and visual field MD. Specifically, the first-line group had a significantly younger mean age, and a higher mean pretreatment IOP and visual field MD compared to the second-line group (Figure 1).

### 3.2. Treatment Outcomes

Table 2 presents the results of the linear mixed-effects model, which analyzed factors influencing the IOP reduction rate following SLT. Significant factors included time (β = −0.29, S.E. = 0.04, t = −7.18, *p* < 0.001), age (β = 0.08, S.E. = 0.04, t = 2.03, *p* = 0.044), pretreatment IOP (β = −1.92, S.E. = 0.21, t = −9.01, *p* < 0.001), and CCT (β = 0.08, S.E. = 0.02, t = 5.32, *p* < 0.001).

The variable “Time” (referring to follow-up timepoints over the 2-year period) was a significant factor influencing SLT-induced IOP reduction. Comparing the first-line and second-line groups, no significant difference was seen in IOP reduction rate. The interaction term “Time*Group” was also not significant, indicating that the reduction in IOP over time was similar in both groups. Therefore, both first-line and second-line SLT were found to significantly reduce IOP in NTG patients over the 2-year follow-up period. The model also showed that patients who were older, had lower baseline IOP values, or had thicker CCT experienced a smaller reduction in IOP following their SLT.

**Figure 2:**
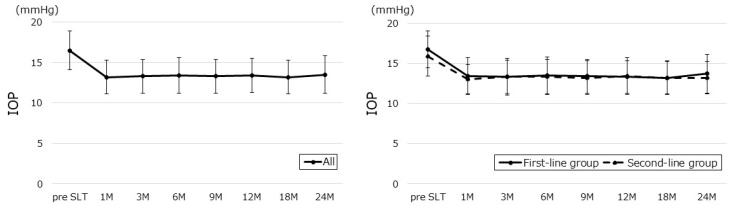
Changes in IOP values before and after SLT. All continuous data are expressed as mean ± standard deviation. IOP, intraocular pressure; SLT, selective laser trabeculoplasty.

**Figure 3:**
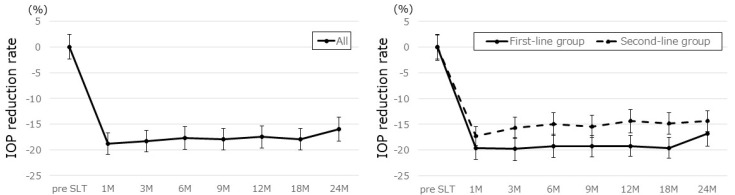
Changes in IOP reduction rate before and after SLT. All continuous data are expressed as mean ± standard deviation. IOP reduction rate (%) = (IOP before SLT − IOP after SLT)/(IOP before SLT) × 100, IOP, intraocular pressure; SLT, selective laser trabeculoplasty.

The changes in IOP values and the IOP reduction rates before and after SLT are depicted in Figure 2 and Figure 3. Overall, the mean IOP of 16.5 ± 2.4 mmHg before SLT decreased by 17.5 ± 2.1%, reaching 13.4 ± 2.1 mmHg at 12 months, and decreased by 16.0 ± 2.3% to 13.5 ± 2.3 mmHg at 24 months. The mean baseline IOP in the first-line group of 16.7 ± 2.3 mmHg dropped by 19.2 ± 2.0%, to 13.3 ± 2.0 mmHg at 1 year, and decreased by 16.8 ± 2.4% to 13.7 ± 2.4 mmHg at 2 years. The mean baseline IOP in the second-line group of 15.9 ± 2.5 mmHg was reduced by 14.4 ± 2.3% to 13.4 ± 2.3 mmHg after 1 year, and by 14.4 ± 2.0% to 13.2 ± 2.0 mmHg after 2 years.

**Figure 4:**
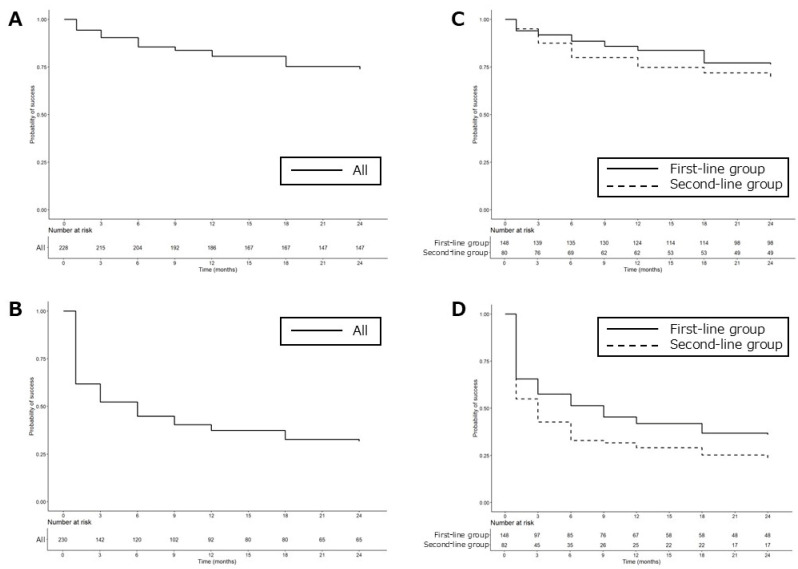
Success rates according to Kaplan–Meier survival analyses for definition A (**A**,**C**) and definition B (**B**,**D**). Success for definition A was defined as a ΔOP ≥ 20%, and for definition B was an IOP reduction rate ≥ 20% without any further IOP-lowering medications, no repeat SLT, nor any additional glaucoma surgeries. ΔOP (%) = (IOP before SLT − IOP after SLT)/(IOP before SLT − 10) × 100, IOP reduction rate (%) = (IOP before SLT − IOP after SLT)/(IOP before SLT) × 100, ΔOP, outflow pressure improvement rate; IOP, intraocular pressure; SLT, selective laser trabeculoplasty.

According to the Kaplan–Meier survival analysis, definition A (ΔOP ≥ 20%) had a 73.7% success rate (Figure 4A) and definition B (IOP reduction rate ≥ 20%) had a 31.6% success rate (Figure 4B) at 24 months. Viewed by group at the 2-year mark, the first-line group had a success rate of 76.3% for definition A and 36.1% for definition B, while the second-line group had success rates of 69.0% for definition A and 23.7% for definition B (Figure 4C,D).

Treatment failure risk factors were evaluated using a Cox proportional hazards regression model (Table 3). Both univariate and multivariate analyses were performed, examining SLT type and baseline variables including age, pretreatment IOP, visual field MD, and CCT. For definition A (ΔOP ≥ 20%), CCT emerged as a significant predictor of failure, with an adjusted hazard ratio (HR) of 1.015 (95% CI: 1.005–1.024, *p* = 0.003). The other variables of SLT type, age, pretreatment IOP, and visual field MD were not significantly associated with failure in the multivariate model. Under definition B (IOP reduction rate ≥ 20%), both a higher CCT (adjusted HR 1.013, 95% CI: 1.006–1.020, *p* < 0.001) and a lower baseline IOP (adjusted HR 0.788, 95% CI: 0.719–0.864, *p* < 0.001) were significantly associated with lower treatment success over 2 years. In contrast, SLT type, age, and visual field MD did not significantly influence the outcome in the multivariate model.

### 3.3. Complications

Table 4 shows SLT-associated adverse events observed from 60 min after the procedure to the end of the 2-year follow-up period. No cases of post-laser IOP spike (>5 mmHg from pretreatment IOP) were observed at either 1 h or 1 week after SLT. Anterior chamber inflammation was reported in 141 eyes (61.3%) during SLT and 18 eyes (7.8%) after SLT, and conjunctival hyperemia was reported in 37 eyes (16.1%) during SLT and 19 eyes (8.3%) after SLT. These symptoms disappeared within a week. Macular edema due to branch retinal vein occlusion occurred after SLT in one eye, which we reported on previously [26]. Endothelial cell density showed no statistically significant change at 2 years after SLT (2635.2 ± 295.6/mm^2^ before SLT and 2682.9 ± 308.5/mm^2^ at 2 years after SLT, *p* = 0.580, paired *t*-test). No severe adverse SLT-associated events were observed at the 24-month mark.

## 4. Discussion

Since the publication of the LiGHT trial in 2019, which demonstrated that SLT is effective as a first-line treatment for POAG and OH [13,14], SLT has gained attention in Japan as a first-line treatment for OAG or as a second-line option for patients concerned about adverse events or adherence issues due to multiple medications. However, most Japanese patients with OAG are diagnosed with NTG, and there is only limited evidence regarding the effectiveness of SLT for NTG. Additionally, it has been reported that first-line SLT in cases of POAG and OH produce a greater IOP reduction in patients with a higher pretreatment IOP, and less in those with a lower pretreatment IOP [27]. To support broader use of SLT in clinical practice in Japan, more data on its long-term efficacy, associated risk factors, and safety in NTG patients are required. Thus, this collaborative multicenter study was conducted under the support of the Japan Glaucoma Society.

One of the key findings in this study is that IOP reductions were maintained over the two-year follow-up period. The IOP reduction after SLT was similar between the two treatment groups. No severe adverse events were observed during the two years following SLT, and most adverse events were minor and transient. In contrast to our previous study, which had a one-year follow-up period [26], and other studies with small sample sizes [24,25], this longer follow-up and larger sample size provide more robust evidence and contribute to a better understanding of the efficacy of SLT in NTG. Another important finding in the current study was the association between pretreatment IOP and CCT with the efficacy of SLT. The clinical significance of these findings is reinforced by the relatively large sample of NTG eyes included in the study.

Previous reports [24,25] have demonstrated that SLT can achieve a meaningful and sustained reduction in IOP, even in patients with NTG. In a study by Nitta et al. [24], 40 Japanese NTG patients received SLT as a first-line therapy, resulting in a significant IOP decrease from 15.8 mmHg to 13.5 mmHg (a 12.7% reduction) at three years post-treatment. Similarly, in our study, the baseline IOP in the first-line SLT group was 16.7 mmHg and declined by 16.8% to 13.7 mmHg at two years, which supports their findings [24].

However, this level of IOP reduction, while statistically significant, may not be sufficient from a clinical standpoint. According to the Collaborative Normal-Tension Glaucoma Study [3,28], a 30% reduction in IOP was necessary to slow visual field deterioration in NTG, suggesting that SLT alone might fall short of the ideal therapeutic target. That said, a long-term study by Kashiwagi et al. [29] examined the efficacy of latanoprost monotherapy in 72 Japanese OAG eyes (65% NTG, 35% POAG), and showed IOP reductions ranging from 10.6% to 15.5% over five years, with stable visual fields maintained in 70% of cases. These outcomes are broadly comparable to the IOP-lowering effects observed with first-line SLT in our study.

Beyond lowering IOP, SLT may offer additional benefits by reducing diurnal IOP fluctuations and enhancing treatment adherence and quality of life (QOL) [30,31,32,33,34], factors that may influence NTG progression. Poor adherence to glaucoma medication is a persistent challenge, particularly in older adults who may face age-related declines in dexterity and cognition [35].

This issue is especially relevant in Japan, where the elderly population continues to grow. As of the latest national statistics [36], individuals aged 65 or older account for 29.1% of the population, with those aged 75 and above comprising 16.1%. For some elderly patients, using eye drops can be difficult due to physical limitations or cognitive decline. The LiGHT trial reported better adherence and QOL outcomes in patients treated with SLT compared to those using eye drops. Consequently, SLT may contribute to improved compliance and QOL by reducing the reliance on topical medications in glaucoma management.

In cases where glaucoma is insufficiently controlled with an initial IOP-lowering eye drop, additional medications are typically introduced to manage IOP. However, using multiple medications simultaneously can increase the risk of side effects. Moreover, in elderly patients, adherence may decline when multiple eye drops are prescribed. For such individuals, further IOP reduction with SLT may offer benefits by minimizing adverse effects from polypharmacy and reducing the number of required medications. Broadway et al. [37] previously indicated that SLT might delay or even eliminate the need for more intensive drug regimens or surgical intervention, potentially at a lower cost. Based on these considerations, we included patients who underwent SLT as a second-line treatment in this study.

In the second-line group, the mean IOP decreased from 15.9 mmHg before SLT to 13.2 mmHg at 24 months, reflecting a 14.4% reduction. A similar study by Lee et al. [25] found that in medicated NTG eyes (using an average of 1.5 glaucoma medications before SLT), IOP decreased from 16.2 mmHg to 12.6 mmHg at 24 months, a 22.0% reduction. Although direct comparisons are limited due to differences in concomitant medication use, our findings similarly suggest that SLT even as a second-line therapy can substantially reduce IOP in NTG patients.

In evaluating the efficacy of SLT in NTG patients, who inherently have low baseline IOP, we considered it inappropriate to assess treatment success based solely on whether the IOP was reduced by 20% or more. Therefore, to more accurately evaluate the IOP-lowering effect in this patient population, we adopted ΔOP as an outcome measure and defined treatment success as achieving a ΔOP of 20% or greater (definition A). As the duration of IOP control is just as important as the extent of reduction, especially in laser treatments, we conducted a Kaplan–Meier survival analysis to evaluate the long-term effectiveness of SLT. Based on definition A (ΔOP ≥ 20%), the success rate was 73.7% at two years (Figure 4A). Given that 10–20% of patients may not respond to SLT, these results suggest that the majority of NTG patients who initially respond to SLT maintain therapeutic benefit for at least two years. We also set the EVP at 10 mmHg in accordance with an earlier study [38]. Since SLT efficacy could be underestimated in eyes with a baseline IOP close to EVP, we excluded patients with a pretreatment IOP < 14 mmHg. In contrast, using definition B (IOP reduction rate ≥ 20%), the success rate was low, 31.6% at two years (Figure 4B). A similar trend was observed by Lee et al. [25], who reported a 2-year success rate of just 11.1% using the same definition in medicated NTG patients. These findings suggest that ΔOP may be a more suitable indicator for evaluating SLT effectiveness in NTG than a percentage-based reduction.

When comparing the first-line and second-line SLT groups, the first-line group demonstrated a higher success rate at two years (Figure 4C,D). One possible explanation is that the first-line group had significantly higher baseline IOP than the second-line group (Table 1). Our results support prior studies in POAG and OHT patients [13,14,15,16,17], showing that eyes without any prior medical treatment are more likely to have a favorable response to SLT than eyes already receiving glaucoma medications.

In this study, we confirmed that both first-line and second-line SLT treatments achieved sustained IOP reductions over a period of at least two years. This was made possible by extending the observation period and enrolling more than twice the number of eyes compared to our previous report. These findings are valuable for both clinicians and patients with NTG when making informed decisions about SLT use in real-world clinical settings.

However, another key consideration is the ability to predict treatment outcomes. Identifying predictors of SLT success is essential for determining its suitability for NTG patients. Although SLT may be more effective when performed before initiating multiple IOP-lowering eye drops, it can be a difficult choice for patients compared to topical therapy. Without reliable indicators to forecast treatment efficacy, clinicians may hesitate to recommend SLT as an initial or secondary intervention.

Our analysis showed that a lower baseline IOP and thicker CCT were associated with a higher risk of treatment failure. On the other hand, whether SLT was used as a first- or second-line treatment did not significantly influence success rates based on either evaluation definition. Previous research has indicated that SLT tends to achieve greater IOP reductions in patients with higher pretreatment IOP and smaller reductions in those with lower IOP, especially in POAG and OH cases [27,39,40,41].

In terms of NTG, Lee et al. [42] reported that a higher baseline IOP predicted better outcomes one month after SLT in 60 eyes receiving medications. Similarly, our results from 230 NTG eyes showed a correlation between a lower baseline IOP and reduced treatment effectiveness, consistent with earlier findings. These results support the view that baseline IOP is a critical factor influencing SLT outcomes, not only in POAG but also in NTG.

Interestingly, some predictors of SLT response may differ between NTG and POAG. In our NTG cohort, a thicker CCT was a significant predictor of treatment failure. CCT is known to influence IOP measurements obtained via applanation tonometry: thicker corneas can overestimate IOP, while thinner ones can underestimate it [43,44]. This suggests that the actual IOP may have been lower than that measured in patients with thicker corneas. In such cases, especially among NTG patients whose IOPs are already within the statistically normal range, the potential for further IOP reduction from SLT may be limited. This could explain why thicker CCT emerged as a risk factor for poor treatment response in our NTG sample.

Importantly, this does not necessarily mean that SLT is intrinsically less effective in eyes with thicker corneas. Instead, the reduced apparent efficacy may stem from overestimated baseline IOP values due to corneal thickness, making true IOP reductions appear smaller. In contrast, the LiGHT trial found that even though IOP reductions decreased with increasing CCT and increased with decreasing CCT, there was no statistically significant correlation between CCT and IOP reduction after SLT in POAG and OH patients without a history of prior IOP-lowering treatment [27].

We hypothesize that CCT may have a more noticeable influence in NTG compared to POAG or OH because of the lower baseline IOP typical of NTG. This difference in initial IOP levels likely contributes to the divergent role CCT plays as a predictor of SLT effectiveness across glaucoma subtypes.

No serious adverse events were observed in this study, which had a longer follow-up period and more than twice the number of participants compared to our previous research [26]. Although several complications have been reported following SLT, such as transient IOP spikes, anterior chamber hemorrhage, iritis, macular edema (ME), and corneal edema [45,46,47,48,49,50,51], none were serious in our cohort. One patient developed ME due to macular BRVO, but the condition resolved without treatment within three months, and no causal link with SLT could be established [26]. While previous studies have noted a temporary decline in corneal endothelial cell density (ECD) following SLT [47,52], no significant change in ECD was found at either 12 months [26] or 24 months after treatment in this study.

Among the mild SLT-related side effects observed, anterior chamber inflammation was the most common (61.3%), followed by conjunctival hyperemia (16.1%) and ocular discomfort (8.3%). However, these symptoms were transient and all resolved within a week. Based on these findings, SLT can generally be regarded as a safe therapeutic option for glaucoma management.

This study has several limitations. First, although SLT may offer sustained IOP reduction in NTG, the follow-up period was limited to two years. Longer-term studies are needed to assess the durability of SLT’s effects. Second, the absence of a control group and differences in baseline characteristics between the first-line and second-line treatment groups may limit the generalizability of our findings.

In summary, SLT was found to be a safe and effective option for both treatment-naïve and previously medicated patients with NTG over a two-year period. However, the extent of IOP reduction was less in eyes with a lower baseline IOP. Moreover, thicker central corneas may lead to an underestimation of SLT efficacy in NTG. Further research is warranted to confirm these observations and determine the long-term benefits of SLT in this patient population.

## Figures and Tables

**Table 1:** Baseline characteristics.

Parameter	All	First-Line	Second-Line	*p*-Value
Eyes (n)	230	148	82	
Age (years)	60.8 ± 11.9	59.1 ± 11.7	64.0 ± 11.7	0.003 *
Gender (female/male)	143/87	96/52	47/35	0.261 ^†^
Pretreatment IOP (mmHg)	16.5 ± 2.4	16.7 ± 2.3	15.9 ± 2.5	0.016 *
Visual field, MD (dB)	−4.3 ± 4.2	−3.8 ± 4.2	−5.0 ± 4.0	0.035 *
Refractive error (spherical D)	−3.91 ± 3.66	−4.04 ± 3.75	−3.68 ± 3.51	0.468 *
Decimal visual acuity	1.18 ± 0.26	1.19 ± 0.26	1.18 ± 0.27	0.804 ^‡^
CCT (μm)	531.6 ± 31.7	533.0 ± 33.5	529.0 ± 28.3	0.331 *
ECD (/mm^2^)	2635.2 ± 295.6	2653.1 ± 296.0	2603.2 ± 293.9	0.222 *

Mean ± standard deviation, * Welch Two Sample *t*-test; ^†^ Fisher’s exact test; ^‡^ Wilcoxon rank sum test, IOP, intraocular pressure; MD, mean deviation; CCT, central corneal thickness; ECD, endothelial cell density.

**Table 2:** Linear mixed-effects model for factors affecting IOP reduction rate after SLT.

	β	S.E.	t Value	*p*-Value
Time (months)	−0.2872	0.0400	−7.1800	0.0000 *
Group (second-line)	1.0163	1.1707	0.8680	0.3859
Age	0.0840	0.0413	2.0310	0.0435 *
Pretreatment IOP	−1.9169	0.2126	−9.0140	0.0000 *
CCT	0.0846	0.0159	5.3210	0.0000 *
Time*Group (second-line)	0.0706	0.0679	1.0400	0.2984

IOP reduction rate (%) = (IOP before SLT − IOP after SLT)/(IOP before SLT) × 100, Patient ID was set as a random effect, while Time (visit timepoints during 2 years after SLT), Group (SLT type), Age, Pretreatment IOP, CCT, and Time*Group (the interaction term between visit timepoints and SLT type) were included as fixed effects. The variance of the intercept by ID, included as a random effect, was 38.1. IOP, intraocular pressure; CCT, central corneal thickness, * represents statistically significance (*p*-value < 0.05).

**Table 3:** Risk factors for success of SLT with definitions A and B.

Univariate Model	HR	95% CI		*p*-Value
		Lower Limit	Upper Limit	
**Group**				
Definition A	1.408	0.824	2.405	0.211
Definition B	1.534	1.047	2.246	0.028 *
Age				
Definition A	1.007	0.985	1.029	0.547
Definition B	1.018	1.002	1.035	0.031 *
Pretreatment IOP				
Definition A	1.043	0.932	1.168	0.465
Definition B	0.825	0.759	0.896	0.000 *
Visual field MD				
Definition A	0.980	0.922	1.043	0.531
Definition B	0.998	0.955	1.043	0.931
CCT				
Definition A	1.012	1.004	1.021	0.006 *
Definition B	1.005	0.999	1.011	0.090
**Multivariate model**	**Adjusted HR**	**95% CI**		***p*-value**
		**Lower limit**	**Upper limit**	
**Group**				
Definition A	1.451	0.829	2.538	0.192
Definition B	1.458	0.969	2.195	0.071
Age				
Definition A	1.007	0.984	1.030	0.567
Definition B	1.013	0.995	1.031	0.149
Pretreatment IOP				
Definition A	1.004	0.887	1.137	0.952
Definition B	0.788	0.719	0.864	0.000 *
Visual field MD				
Definition A	0.967	0.907	1.030	0.295
Definition B	0.996	0.949	1.046	0.876
CCT				
Definition A	1.015	1.005	1.024	0.003 *
Definition B	1.013	1.006	1.020	0.000 *

SLT, selective laser trabeculoplasty; HR, hazard ratio; CI, confidence interval; IOP, intraocular pressure; MD, mean deviation; CCT, central corneal thickness, *p*-values were calculated by Cox proportional hazard regression model, * represents statistical significance (*p*-value < 0.05).

**Table 4:** Summary of SLT-related adverse events.

Adverse Events During SLT	Eyes (n)	Incidence Rate (%)
Anterior chamber inflammation	141	61.3
Conjunctival hyperemia	37	16.1
**Adverse events after SLT**	**Eyes (n)**	**Incidence rate (%)**
Conjunctival hyperemia	19	8.3
Discomfort (ocular or headache)	19	8.3
Anterior chamber inflammation	18	7.8
Blurred/altered vision	14	6.1
Photophobia	5	2.2
Macular edema	1 (Due to BRVO)	0.4

SLT, selective laser trabeculoplasty; BRVO, branch retinal vein occlusion.

## Data Availability

The data that support the findings of this study are available from the corresponding author upon reasonable request.
